# Longitudinal plasma interleukin‐6 and post‐stroke cognitive outcomes: The Stroke‐IMPaCT study

**DOI:** 10.1002/alz.71261

**Published:** 2026-03-10

**Authors:** Natasha S. Carmichael, Harry R. Deijnen, Siew Yan Wong, Thomas O. Williams, Evangelos Kontopantelis, Luke Cowie, Eileen Jones, Lauren Drag, Marion S. Buckwalter, John R. Grainger, Stuart M. Allan, Craig J. Smith

**Affiliations:** ^1^ Lydia Becker Institute of Immunology and Inflammation Faculty of Biology Medicine and Health The University of Manchester Manchester UK; ^2^ Division of Cardiovascular Sciences The University of Manchester Manchester UK; ^3^ Geoffrey Jefferson Brain Research Centre Manchester Academic Health Science Centre Northern Care Alliance NHS Foundation Trust University of Manchester Manchester UK; ^4^ Manchester Centre for Clinical Neurosciences Northern Care Alliance NHS Foundation Trust Salford UK; ^5^ Division of Immunology Immunity to Infection and Respiratory Medicine The University of Manchester Manchester UK; ^6^ Division of Informatics Imaging and Data Sciences The University of Manchester Manchester UK; ^7^ Division of Family Medicine Yong Loo Lin School of Medicine National University of Singapore Singapore; ^8^ Department of Neurology and Neurological Sciences Stanford School of Medicine Stanford California USA; ^9^ Stanford Stroke Centre Stanford School of Medicine Stanford California USA; ^10^ Department of Neurosurgery Stanford School of Medicine Stanford California USA; ^11^ Division of Neuroscience The University of Manchester Manchester UK

**Keywords:** interleukin‐6, inflammation, ischemic stroke, post‐stroke cognition, smoking, socioeconomic status

## Abstract

**INTRODUCTION:**

Inflammatory factors, particularly interleukin (IL)‐6, are implicated in post‐stroke cognitive decline, yet the association with longitudinal changes in these markers remains unclear.

**METHODS:**

Plasma IL‐6 and other inflammatory markers were measured within 96 hours of ischemic stroke, and at 6–9 and 18–21 months, alongside cognitive assessment. Associations between inflammatory factors and cognition were examined using adjusted regression models.

**RESULTS:**

A doubling of IL‐6 between admission and 6–9 months was associated with cognitive impairment at 18–21 months (odds ratio [OR] = 8.16; 95% confidence interval [CI] 1.82–47.26; *p *= 0.01), while each one‐unit IL‐6 increase was linked to a 1.5‐point decrease in memory Z‐scores (β = ‐1.50; 95% CI ‐2.57–0.43; *p *= 0.007). Smokers showed persistently blunted IL‐6 trajectories (*p *< 0.05) and downregulated Toll‐like receptor signaling (*p *< 0.05). Exploratory analyses suggested that lower socioeconomic status may relate to 6‐month IL‐6 concentrations via smoking.

**DISCUSSION:**

Post‐stroke IL‐6 trajectories associate with later cognition, highlighting potential therapeutic targets.

## Highlights

- Rising interleukin (IL)‐6 by 6–9 months post‐ischemic stroke relates to worse long‐term cognition.
- This association is stronger than that seen with admission IL‐6 levels.
- Exploratory analyses suggest smoking and higher deprivation may drive elevated IL‐6.
- Smoking reduces Toll‐like receptor (TLR) signaling, strengthening a link with post‐stroke IL‐6 levels.
- Subacute circulating IL‐6 changes may predict or enable interventions for cognition.

## 1 BACKGROUND

Post‐stroke cognitive decline (PSCD) is a serious complication of ischemic stroke, posing significant burdens on both affected individuals and healthcare systems. Recent meta‐analyses and cohort studies estimate that PSCD affects 40‐50% of stroke survivors within the first year.^1^, ^2^, ^3^, ^4^ The incidence of PSCD is influenced by stroke severity, affecting ∼34% of patients with severe strokes (National Institute of Health Stroke Scale (NIHSS) score > 10) within one‐year post‐stroke, 8% with minor strokes (NIHSS score < 3), and even 5% in cases of transient ischemic attacks (TIAs).^5^ While the initial severity and location of the stroke are important contributors, they do not fully explain long‐term cognitive outcomes, and the precise mechanisms underlying PSCD remain elusive. PSCD has a biphasic onset, whereby changes in the initial six months post‐stroke show associations with stroke‐specific factors, but later cognitive changes are unrelated to stroke size and location.^5^ Further, some individuals remain cognitively stable for several years after a stroke before developing PSCD or progressing to dementia.^6^, ^7^ Together, these patterns point to a complex and multifactorial pathophysiology for PSCD, in which stroke is the initiating event, but not the sole driver of later cognitive deterioration.

Growing evidence highlights the peripheral immune system as a key mediator of PSCD.^8^, ^9^, ^10^ The acute phase of ischemic stroke is characterized by marked immune activation,^11^, ^12^, ^13^, ^14^ with elevated circulating interleukin (IL)‐6 representing the most well‐established hallmark of the response.^15^, ^16^, ^17^, ^18^ After ischemic stroke, IL‐6 correlates with infarct volume,^19^ stroke severity,^20^ and poorer functional outcomes,^21^, ^22^, ^23^, ^24^ with emerging studies suggesting associations with subsequent cognitive impairment.^9^, ^25^, ^26^ Further, other inflammatory mediators have also been reported to change in the acute phase of ischemic stroke, including C‐reactive protein (CRP), tumor necrosis factor‐alpha (TNF‐α), chemokine ligand 3 (CCL3), and matrix metalloproteinases (MMPs), and similarly demonstrate associations with clinical parameters and patient outcomes.^27^, ^28^, ^29^, ^30^ Most investigations, however, have focused on single measurements of circulating factors acutely, or in the early post‐ischemic stroke period, potentially overlooking dynamic changes over time. Since immune–cognition relationships are unlikely to be static, characterizing longitudinal trajectories of inflammatory factors may provide critical insights into their relationships with PSCD.

Additionally, how immune responses are shaped by demographic factors requires necessary consideration. Stroke patients frequently present with comorbidities such as diabetes and hypertension,^31^ which can alter inflammatory pathways and generate distinct immune environments even before stroke onset.^32^, ^33^ Socioeconomic status may exert similar influences; high deprivation has been linked to persistently elevated inflammatory markers,^34^, ^35^ while early‐life disadvantage is associated with higher CRP in adulthood.^36^ Overall, the potential effects of these contextual factors may shape both immune phenotypes and vulnerability to stroke and PSCD.

### 1.1 Study aims

Our overarching aim was to characterize circulating inflammatory factor concentrations longitudinally after ischemic stroke, rather than relying on single acute phase measurements, and investigate their relationships with cognition. Specifically, we focused on IL‐6 given its previous associations with post‐stroke outcomes, but eight additional factors (CCL2, CCL3, CD163, CRP, IL‐1 receptor antagonist [IL‐1Ra], IL‐18, IL‐8 and MMP‐9) were selected for their known inflammatory roles and prior associations with stroke.^9^, ^37^, ^38^, ^39^, ^40^, ^41^, ^42^, ^43^ To examine inflammation over a timescale relevant for PSCD,^5^ we evaluated inflammatory factors < 96 hours after ischemic stroke, as well as at 6–9 and 18–21 months post‐stroke, in participants who also received a comprehensive cognitive battery at the same follow‐up timepoints.

**RESEARCH IN CONTEXT**

1. **Systematic review**: The authors reviewed existing literature using PubMed searches, combining terms such as “inflammatory factors”, “post‐stroke cognition”, and “interleukin‐6” (IL‐6). Prior research linking circulating inflammatory factors to post‐stroke cognition is appropriately cited, yet few studies have examined longitudinal IL‐6 changes.
2. **Interpretation**: Our findings reveal a previously unappreciated concept that changes in plasma IL‐6 concentrations within the first 6–9 months post‐ischemic stroke are independently associated with later cognition. These associations are stronger than for admission IL‐6 concentrations. In exploratory analyses, smoking and socioeconomic status emerged as potential modifiers of circulating IL‐6, suggesting links between social factors, biological recovery and cognition.
3. **Future directions**: The manuscript identifies IL‐6 trajectories as a key indicator of post‐stroke cognitive outcome and supports further investigation into mechanisms linking IL‐6 to post‐stroke cognitive decline and the potential application of IL‐6–targeted interventions. Future work should focus on defining the optimal therapeutic window for IL‐6 antagonists in post‐stroke treatment.

The objectives of the study were to: (1) Assess plasma IL‐6 and other immune markers longitudinally across multiple timepoints following ischemic stroke, compared to non‐stroke vascular‐risk controls, using a high‐precision immunoassay platform (the Ella system), to enable sensitive and quantitative detection across a broad dynamic range; (2) evaluate the relationships between plasma IL‐6 and other inflammatory factors (CCL2, CCL3, CD163, CRP, IL‐1Ra, IL‐18, IL‐8, MMP‐9), with comprehensive longitudinal cognitive outcomes, including domain‐specific metrics; (3) incorporate socioeconomic and clinical factors to evaluate how contextual influences may modulate immune function and cognitive outcomes.

## 2 METHODS

### 2.1 Study design

**FIGURE 1:**
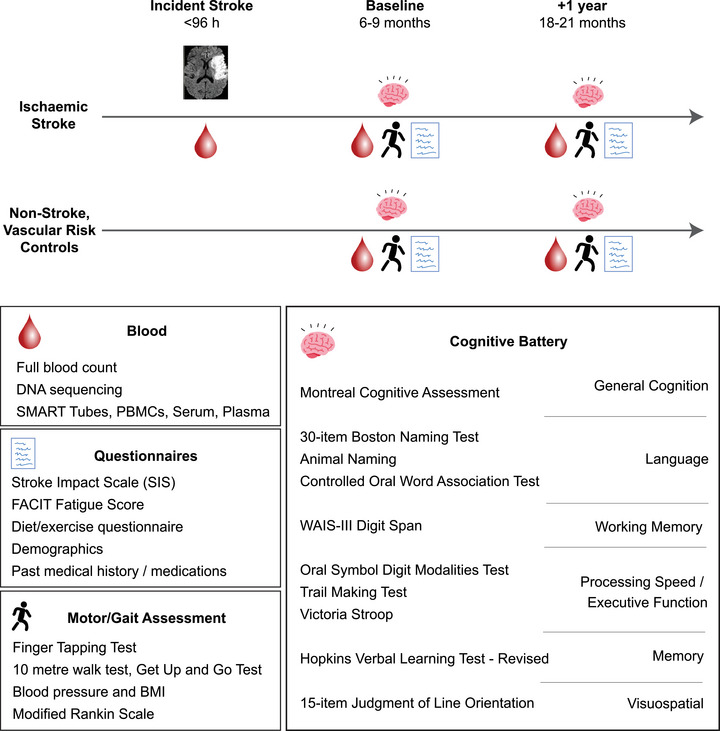
Stroke‐IMPaCT Study design. Acute ischemic stroke patients recruited to the study had clinical and demographic information recorded upon admission, alongside cognitive screening (MoCA) and venous blood collection within 96 hours of stroke onset or time last seen well. Patients were invited to return for follow‐up visits at 6‐ to 9‐month and 18‐ to 21‐month timepoints, consisting of demographic information collection, venous blood sampling and cognitive assessment. Cognitive outcomes at follow‐up visits were evaluated via a detailed battery of neurological testing. Non‐stroke, vascular‐risk controls refer to age‐ and sex‐matched individuals with no history of stroke or significant cognitive impairment, but with established vascular disease or two or more vascular risk factors. MoCA, Montreal Cognitive Assessment; Stroke‐IMPaCT, Stroke‐Immune Pathways and Cognitive Trajectory.

The Stroke‐Immune Pathways and Cognitive Trajectory (Stroke‐IMPaCT) Study is an ongoing, longitudinal, prospective, multi‐center study undertaken at stroke centers in the United Kingdom (Manchester) and the United States of America (Stanford and North Manhattan) (Figure 1), investigating relationships between post‐stroke peripheral immune status and cognitive trajectories.

The present paper reports only on participants from the Manchester site who were enrolled and tested up to September 2025, comprising 190 ischemic stroke patients assessed at admission and 48 non‐stroke, vascular‐risk controls. These were the only study participants with available socioeconomic data. Regulatory approvals were obtained from the Wales Research Ethics Committee (REC) (REC reference 21/WA/0156; Integrated Research Application System [IRAS] ID 275726).

### 2.2 Stroke participant eligibility and consent

Inclusion criteria: Patients with ischemic stroke confirmed by brain imaging (computed tomography [CT], magnetic resonance imaging [MRI], or both); aged 45 years or over; able to undergo venous blood sampling within 96 hours of stroke symptom onset; sufficiently fluent in English and able to return for follow‐up visits.

Exclusion criteria: Score of two points or more on the language component of the NIHSS (indicating significant aphasia); pre‐existing neurological, psychiatric, or other condition (e.g., blindness) that would make it difficult to accurately assess cognitive outcomes; history of previous symptomatic hemorrhagic stroke; deemed unlikely to survive to follow‐up or palliative care considered to be imminent; established diagnosis of dementia predating the stroke; informed consent not available.

Consecutive admissions to the Comprehensive Stroke Centre, Manchester Centre for Clinical Neurosciences (MCCN) with confirmed ischemic stroke were screened for eligibility by trained research practitioners. Following confirmation of eligibility and provision of study information material, informed consent was obtained. If a potential participant was assessed as lacking capacity to provide written informed consent because of reduced conscious level, cognitive or communication problems, then consent was obtained from a personal consultee.

### 2.3 Non‐stroke, vascular‐risk control participant eligibility

Potential control participants without a previous history of stroke or dementia were identified through the National Institute for Health and Care Research (NIHR) Research for the Future program (https://researchforthefuture.org/about‐us/).

Inclusion criteria: Aged 45 years or over; at least two vascular risk factors^†^ of any duration or one vascular risk factor for at least 5 years and/or established vascular disease^#^; sufficiently fluent in English; living independently in the community; willing and able to give consent to study participation.

Exclusion criteria: History of any previous stroke, TIA, significant head injury, autoimmune neurological disease, current brain tumor, current nervous system infection or neurodegenerative disease; undergoing treatment (now or within the preceding 6 weeks) for autoimmune, oncological or infectious disease; pre‐existing neurological, psychiatric, or other condition (e.g., blindness) that would make it difficult to accurately assess cognitive outcomes; currently participating in a clinical trial of an investigational medicinal product (CTIMP) or device trial; within 3 months of an acute ischemic event (e.g., myocardial infarction) or major surgery (e.g., coronary artery bypass grafts).

*^†^Vascular risk factors*: treated hypertension; diabetes mellitus; treated hyperlipidemia; current smoker; confirmed atrial fibrillation; confirmed left ventricular hypertrophy; raised body mass index > 25.

*^#^Established vascular disease*: coronary artery disease (defined as any of: treated angina; previous myocardial infarction or acute coronary syndrome more than three months ago; coronary artery bypass or stents more than 3 months ago) or peripheral vascular disease (defined as any of: intermittent claudication; neuroischaemic ulceration; previous surgical bypass or endovascular treatment more than 3 months ago; amputation more than three months ago; abdominal aortic aneurysm; renal artery stenosis).

After completing a screening questionnaire, potential participants were pre‐screened using the telephone version of the Montreal Cognitive Assessment (MoCA).^44^ Potential participants scoring 19 or more points were then invited to attend for confirmation of eligibility, provision of consent and their study visit.

### 2.4 Baseline and follow‐up data collection

Patients’ clinical and demographic information were recorded upon admission, including age, sex at birth, ethnicity, pre‐stroke modified Rankin Scale (mRS) score, baseline NIHSS score, hyperacute treatments (thrombolysis and thrombectomy), known vascular risk factors and past medical history, medications, and smoking and alcohol history. The MoCA was recorded within the first 7 days of admission by trained occupational therapists or research practitioners. Socioeconomic status was determined using the English Index of Multiple Deprivation Postcode Checker (https://www.fscbiodiversity.uk/imd/), an area‐level composite measure of deprivation based on seven domains (income, employment, education, health, crime, housing and services, and living environment), with lower deciles indicating greater deprivation. Each participant's residential postcode was linked to an Index of Multiple Deprivation decile.

Venous blood was drawn from stroke participants within 96 hours of stroke symptom onset or time last seen well. Whenever possible, blood draws were performed between 0800 and 1400. Blood was collected in ethylenediaminetetraacetic acid (EDTA) tubes and processed within 4 hours.

Patients were invited back for follow‐up visits at 6‐ to 9‐month and 18‐ to 21‐month timepoints, with clinical and demographic information collected as prior, alongside blood draws and cognitive assessment. Parallel demographics, vascular risk factors, past medical history, and medications were also recorded, enrolment blood collected, and cognitive assessments performed, for participating non‐stroke controls.

### 2.5 Cognitive assessments

Cognitive performance at 6–9 and 18–21 months was assessed using a 60‐minute comprehensive cognitive battery that included the MoCA and standardized measures of five cognitive domains.^45^ Cognition at study entry solely included the MoCA. All cognitive outcome assessments were undertaken by research staff trained under the supervision of the study neuropsychologist. MoCA scores were adjusted for educational attainment by adding one point for individuals with fewer than 12 years of education. The comprehensive battery comprised nine standardized neuropsychological tests, yielding 13 cognitive variables, as described previously.^45^ In brief, tests were clustered into five cognitive domains based on prior knowledge and a pair‐wise, undirected Pearson correlation graph (t‐distributed stochastic neighbor embedding plot) as follows: language (30‐item Boston Naming Test, Controlled Oral Word Association Test, animal naming), processing speed/executive function (Oral Symbol Digit Modalities Test, Trails A and Trails B, Victoria Stroop Dot, Victoria Stroop Word, Victoria Stroop Color‐Word), visuospatial functioning (15‐item Judgment of Line Orientation), working memory (Wechsler Adult Intelligence Scale‐III Digit Span), and memory (Hopkins Verbal Learning Test‐Revised [HVLT‐R] Immediate Recall, HVLT‐R Delayed Recall). Z‐scores for each domain were calculated by standardizing the raw scores against normative published data matched for age, and in some cases, education (Oral Symbol Digit Modalities Test). A global composite domain score and a global test score were also calculated, with the global domain Z‐score representing the average of Z‐scores across the cognitive domains, and the global test Z‐score reflecting the average of Z‐scores across all individual cognitive tests administered within the battery.

Using the domain‐specific Z‐scores, we defined an a priori binary measure of cognitive status, categorizing individuals as cognitively impaired or unimpaired. Though there is not universal consensus, thresholds of Z < −1 and Z < ‐1.5 are commonly used to classify cognitive impairment,^46^ with trade‐offs in sensitivity and specificity associated with each. For this study, Z‐score ≤ −1 was used to indicate a milder cognitive impairment, reflecting a more liberal threshold, while a Z‐score ≤ ‐1.5 was used to indicate a more moderate cognitive impairment. Following the framework described by Sachdev et al.,^47^ cognitive impairment was defined as either impairment in two or more cognitive domains at the mild threshold (Z‐score ≤ −1) or impairment in at least one cognitive domain at the moderate threshold (Z‐score ≤ −1.5).

### 2.6 Plasma isolation and measurement of inflammatory factors

Plasma was isolated by centrifugation of EDTA blood tubes at 2000 *g* for 10 minutes at 4^0^C. Plasma was aliquoted and stored at ‐80°C until required for further analysis. Following thawing, plasma concentrations of the inflammatory factors of interest (CCL2, CCL3, CD163, CRP, IL‐1Ra, IL‐6, IL‐18, IL‐8 and MMP‐9) were determined using the Ella Automated Immunoassay System (Bio‐Techne, Minneapolis, USA), according to the manufacturer's instructions. Briefly, plasma was diluted in sample diluent (Cat. No. 896098, Bio‐Techne) according to the recommended dilution factor for that analyte, and loaded into Ella cartridges. Wash buffer (Cat. No. 896055, Biotechne) was loaded into the necessary wells, and cartridges were run on the Ella platform.

As the primary analyte of interest, plasma IL‐6 concentrations were measured in *n* = 45 non‐stroke controls; however, other analytes were only measured in *n* = 11 controls, due to funding and resource availability.

### 2.7 Peripheral blood mononuclear cells processing

To obtain peripheral blood mononuclear cells (PBMCs), EDTA blood was diluted 1:1 with phosphate‐buffered saline (PBS) supplemented with 2% fetal bovine serum (FBS) and centrifuged at 1200 *g* for 15 minutes at 20°C in SepMate™ PBMC Isolation Tubes (StemCell Technologies, Vancouver, Canada) with Ficoll‐Paque™ PLUS density gradient media (Cat. No. 17144003; Cytiva Life Sciences, Amersham, UK). Following centrifugation, the top supernatant layer containing PBMCs was transferred to 50 mL Falcon tubes, diluted with 20 mL PBS + 2% FBS and centrifuged at 500 *g* for 10 minutes at 20°C. Supernatant was discarded, and the cell pellet was resuspended in ACK Lysing Buffer (Cat. No. A1049201; Gibco, Waltham, USA) for 5 minutes at room temperature. PBS (10 mL) + 2% FBS was added and tubes centrifuged at 500 *g* for 5 minutes at 20°C. The cell pellet was resuspended in 20 mL FBS + 2% FBS and cells were counted before centrifugation at 500 *g* for 10 minutes at 20°C. Cells were resuspended at a final density of 5‐6 × 10^6^ cells / 500 µL CryoStor® CS10 Freezing Medium (Cat. No. 100‐1061; StemCell Technologies), and frozen at ‐80°C in CoolCell™ Cell Freezing Vial Containers (Cat. No. 15542771; Corning, New York, USA). Finally, PBMC aliquots were transferred to liquid nitrogen until ready for use.

### 2.8 RNA isolation and transcriptomic analyses

Total RNA was extracted from PBMCs using the PureLink RNA Mini Kit (Cat. Nos. 12183018A, 12183020, 12183025; ThermoFisher Scientific), following the manufacturer's protocol. An on‐column DNase digestion step was performed to eliminate residual genomic DNA. RNA was eluted in RNase‐free water, quantified spectrophotometrically (NanoDrop; ThermoFisher), and assessed for integrity using an Agilent Bioanalyzer or TapeStation. Only samples with A260/280 > 1.8 and RNA integrity number (RIN) ≥ 7.0 were included in downstream analyses.

Total RNA was processed for strand‐specific RNA‐seq with rRNA and globin depletion, averaging 20 M paired‐end reads per sample. cDNA libraries (2 × 150 bp) were sequenced on the Illumina NovaSeq™, and raw FASTQ files were processed on the University of Leeds high‐performance computer cluster. Each FASTQ was quality assessed (Fastqc v0.12.1) and trimmed for adapters (Cutadapt v5.0). Mapping and quantification were achieved using Salmon (v1.x) in quasi‐mapping mode to generate transcript‐level abundance estimates (quant.sf files). Gene‐level counts were obtained with the tximport R package (v1.x), summarized using GENCODE v45 annotations. A tx2gene mapping from the GTF file was used to link transcript IDs to gene IDs. Only samples with complete smoking status metadata were included (*n* = 84).

Gene set enrichment analysis was performed using ranked gene lists derived from log_2_ fold‐changes of expressed genes, mapped to Entrez IDs. Genes were collapsed to unique Entrez IDs by selecting the transcript with the highest absolute log_2_ fold‐change. Enrichment testing used the gseKEGG function from the clusterProfiler package.

Analyses were stratified by smoking status (current smoker at the time of stroke versus all others). A priori–selected IL‐6–related inflammatory pathways were evaluated: Toll‐like receptor (TLR), JAK–STAT, cytokine–cytokine receptor interaction, and TNF. Parameters included a minimum gene set size of 10, a maximum of 500, and an adjusted *p* value threshold of 0.05. Results were visualized using enriched pathways and were reported according to significance.

### 2.9 Statistical and data analyses

Baseline demographics, and longitudinal inflammatory factor and cognitive measures, were summarized using appropriate descriptive statistics. Inflammatory factors were log‐transformed using log_10_ to reduce skewness; TLR pathway scores were not transformed as they were normally distributed. IL‐6 was the primary factor of interest, with other analytes considered secondary. To compare inflammatory factor concentrations between ischemic stroke patients and controls, Welch's t‐tests were used. MoCA scores at admission, 6–9 and 18–21 months were compared against the cognitive impairment threshold of 26^44^ using one‐sample Wilcoxon signed‐rank tests. Similarly, domain Z‐scores at 6–9 and 18–21 months were compared against zero to assess deviation from normative cognitive performance. In addition, Mann–Whitney tests were employed to compare Z‐scores from ischemic stroke patients at 6–9 months with those of control participants. All statistical tests were adjusted for multiple comparisons using false discovery rate (FDR) correction.

In this present study, the primary outcome was cognition at 18–21 months. Processing speed/executive function and memory were selected as primary domains of interest, as these were significantly affected in our ischemic stroke cohort. Relationships with global (binary measure of cognitive impairment status) and domain‐specific cognition (continuous Z‐scores) were explored using logistic and linear regression models, respectively. Model assumptions were assessed via several metrics including: variance inflation factors (VIFs) to assess multicollinearity of variables; Shapiro–Wilk tests and Q–Q plots for residual normality; and Breusch–Pagan tests and residual–fitted plots for heteroscedasticity. Where heteroscedasticity was detected, heteroscedasticity‐consistent (HC3) robust standard errors were applied. Logistic regression models were further evaluated using the Hosmer–Lemeshow goodness‐of‐fit test.

As the main analyte of interest, IL‐6 trajectories across three timepoints following ischemic stroke (admission, 6–9 months, and 18–21 months) were analyzed using linear mixed‐effects models (lme4 package). Models included random intercepts for participants to account for within‐subject variability arising from repeated measures. To visualize group‐level patterns, admission IL‐6 concentrations were categorized into terciles (low, medium, high). Model performance was evaluated using marginal and conditional R^2^ values and residual diagnostics.

Mediation analyses were performed in R using the mediation package. Mediator and outcome models were fit using linear regression. Indirect, direct, and total effects were estimated, with 95% bias‐corrected confidence intervals derived from 1000 nonparametric bootstrap simulations.

All models were adjusted for age, sex, stroke severity (NIHSS), hypertension, diabetes, and time of visit (either number of hours post‐stroke for the admission timepoint, or number of days post‐stroke for the follow‐up timepoints). NIHSS was used as the measure of stroke severity, as infarct volume data were only available for *n* = 148 participants, since an MRI scan at admission was not mandatory. Where appropriate, models were additionally adjusted for IL‐6 at admission to capture relative rather than absolute changes. To assess whether pre‐stroke function may account for any of the observed associations between inflammatory factors and cognition, sensitivity analyses were performed by additionally including pre‐stroke mRS score in the regression models. Mediation analyses did not include hypertension or diabetes adjustments due to the limited sample sizes and non‐convergence issues encountered in regression models that included more relevant covariates. Effect modification by pre‐stroke smoking status was tested using interaction terms.

Analyses were conducted using complete case analysis. Participants with missing inflammatory factor or cognitive outcome data at required timepoints were excluded from the relevant models. Multiple imputation was not undertaken due to the small proportion of missing data and the potential to introduce bias given the modest sample size. All statistical tests were two‐tailed, with significance set at *p *< 0.05. FDR correction was applied to secondary analyses as the primary analysis was hypothesis driven.

All analyses were conducted in R (version 4.2.2). Analyses and reporting followed STROBE (Strengthening the Reporting of Observational Studies in Epidemiology) guidelines.

## 3 RESULTS

### 3.1 Study characteristics

All participants were enrolled between August 2021 and July 2025. 190 patients with acute ischemic stroke and 48 non‐stroke, vascular‐risk control participants were recruited at MCCN. Baseline characteristics of the participating patients and controls are presented in Table 1. The ischemic stroke cohort had a median age of 66 (interquartile range [IQR] of 45, 91) years, was predominantly male (70%), had a median NIHSS score of 4 (0, 23), and a median MoCA score of 26 (14, 30) after admission. The ischemic stroke cohort was slightly younger and more male dominant compared to the vascular‐risk control group (median age of 69 years and 54.2% male).

**TABLE 1:** Baseline participant characteristics in the Manchester arm of the Stroke‐IMPaCT Study.

Parameter	Control (*n* = 48)	Stroke (*n* = 190)
Age (years), median (min, max)	69 (48, 82)	66 (45, 91)
**Sex, *n* (%)**		
Male	26 (54.2)	133 (70)
Female	22 (45.8)	57 (30)
**Presentation**		
Onset/Last Seen Well to blood draw, hours, median (IQR)	–	45.3 (25.9)
Right hemisphere, *n* (%)	–	84 (44.2)
Acute infarct volume (mm^3^), median (min, max)^a^	–	2071.5 (69, 81562)
NIHSS, median (min, max)	–	4 (0, 23)
Intravenous thrombolysis, *n* (%)	–	47 (24.7)
Thrombectomy, *n* (%)	–	17 (9.0)
Pre‐stroke mRS score, median (min, max)^b^	–	0 (0, 4)
**Admission risk factors, *n* (%)**		
No. of vascular comorbidities, median (min, max)	3 (0, 6)	2 (0, 6)
Hypertension	32 (66.7)	103 (54.2)
Atrial fibrillation	5 (10.4)	26 (13.7)
Diabetes	21 (43.8)	50 (26.3)
Frequent alcohol consumption^c^	20 (41.7)	37 (19.5)
Smoking status^d^		
Current smoker	2 (4.2)	46 (24.2)
Ex‐smoker	19 (39.6)	33 (17.4)
Never smoker	27 (56.3)	107 (56.3)
Hyperlipidemia	37 (77.1)	91 (47.9)
Coronary artery disease	15 (31.3)	14 (7.4)
**Enrollment medications, *n* (%)**		
No. of medications taken, median (min, max)	4 (0, 9)	2.5 (0, 12)
No. of medication classes taken, median (min, max)	3 (0, 7)	2 (0, 7)
Antiplatelets	19 (39.6)	57 (30.0)
Anticoagulants	3 (6.3)	12 (6.3)
Antidiabetics	19 (39.6)	39 (20.5)
Anti‐depressants	7 (14.6)	19 (10.0)
Antihyperlipidemics	37 (77.1)	91 (47.9)
β‐blocker	16 (33.3)	40 (21.1)
ACE‐inhibitor	16 (33.3)	41 (21.6)
Angiotensin II receptor blocker	8 (16.7)	20 (10.5)
Calcium channel blockers	20 (41.7)	49 (25.8)
Other antihypertensives	5 (10.4)	21 (11.1)
**Differential full blood count (x10^9^/L), median (min, max)** ^e^		
White blood cell count	6.4 (3.9, 11.2)	8.1 (3.1, 16.7)
Neutrophils	3.8 (1.6, 7.8)	5.4 (1.8, 14.3)
Lymphocytes	1.7 (0.7, 3.5)	1.7 (0.6, 7.6)
Monocytes	0.5 (0.3, 2.3)	0.5 (0.2, 1)
Eosinophils	0.1 (0, 0.5)	0.1 (0, 1.5)
Basophils	0 (0, 0.1)	0 (0, 0.3)
Platelets	251 (128, 401)	240 (123, 791)
Admission MoCA score, median (min, max)^f^		
Normed total score	27 (19, 29)	26 (14, 30)

*Note*: All risk factors were defined as a history of treatment for that condition or having been informed by a medical professional. Coronary artery disease was defined as having any of the following: previous myocardial infarct, coronary angioplasty, angina or previous bypass surgery (CABG). Frequent alcohol consumption was defined as more than once a week. Smoking status was defined as follows: current smoker if the individual had smoked in the 30 days prior to stroke; ex‐smoker if they had not smoked in the 30 days prior, but had smoked 100+ cigarettes in their lifetime; never smoker if they fulfilled neither criterion.

Abbreviations: ACE, angiotensin‐converting enzyme; IQR, interquartile range; MoCA, Montreal Cognitive Assessment; mRS, modified Rankin Scale; NIHSS, National Institutes of Health Stroke Scale; Stroke‐IMPaCT, Stroke‐Immune Pathways and Cognitive Trajectory.

The following symbols denote missing data:

^a^ Missing for *n* = 42 stroke patients.

^b^ Missing for *n* = 2 stroke patients.

^c^ Missing for *n* = 1 control and *n* = 27 stroke patients.

^d^ Missing for *n* = 4 stroke patients.

^e^ Missing for *n* = 1 control and *n* = 4 stroke patients.

^f^ Missing for *n* = 2 controls and *n* = 48 stroke patients.

### 3.2 Summary of inflammatory factors longitudinally following ischemic stroke

**FIGURE 2:**
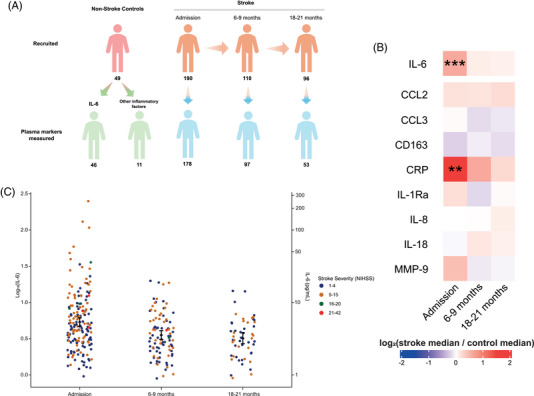
Longitudinal characteristics of circulating inflammatory factors following ischemic stroke. (A) Summary of the number of ischemic stroke patients at each timepoint and non‐stroke controls recruited, and those with inflammatory factors measured, in the Manchester arm of the Stroke‐IMPaCT study. *n* = 45 non‐stroke controls had IL‐6 measured, but *n* = 11 for other analytes. (B) Heatmap showing log_2_ fold change in median plasma inflammatory factor concentrations between ischemic stroke patients at each timepoint versus non‐stroke controls. *p*‐v‐alues were calculated via Welch's *t*‐tests. *** = *p *< 0.001; ** = *p *< 0.01. C) Plasma IL‐6 concentrations across timepoints in ischemic stroke patients. Individual patient measurements are shown as colored points according to standard NIHSS‐defined stroke severity categories: minor (NIHSS 1‐4; blue), moderate (5‐15; orange), moderate–severe (16‐20; green), and severe (21‐42; red). Black diamonds represent estimated marginal means from a linear mixed‐effects model, with error bars indicating 95% confidence intervals. IL, interleukin; NIHSS, National Institute of Health Stroke Scale; Stroke‐IMPaCT, Stroke‐Immune Pathways and Cognitive Trajectory.

Inflammatory factor concentrations were measured in 339 samples in total, from ischemic stroke patients and non‐stroke controls at several timepoints (Figure 2A; Supplementary Table S1). Compared with non‐stroke controls, IL‐6 (t(15.72) = 4.14, *p *< 0.001) and CRP (t(12.20) = 3.16, *p *< 0.01) concentrations were significantly greater acutely following ischemic stroke (Figure 2B).

Given this acute elevation, we examined longitudinal IL‐6 trajectories after ischemic stroke. Plasma IL‐6 concentrations were significantly lower at 6–9 months (*p *< 0.0001) and 18–21 months (*p *< 0.001) compared with admission (Figure 2C; Supplementary Figure S1; Supplementary Table S2), while higher IL‐6 concentrations were associated with greater stroke severity and older age. IL‐6 concentrations at admission and at 6–9 months were not significantly associated with pre‐stroke mRS score, in both adjusted and unadjusted analyses, although a modest, non‐significant trend was observed between IL‐6 concentrations at 6–9 months and pre‐stroke mRS (β = 0.22, 95% CI ‐0.12–0.56; *p *= 0.21).

### 3.3 Summary of cognitive measures

**FIGURE 3:**
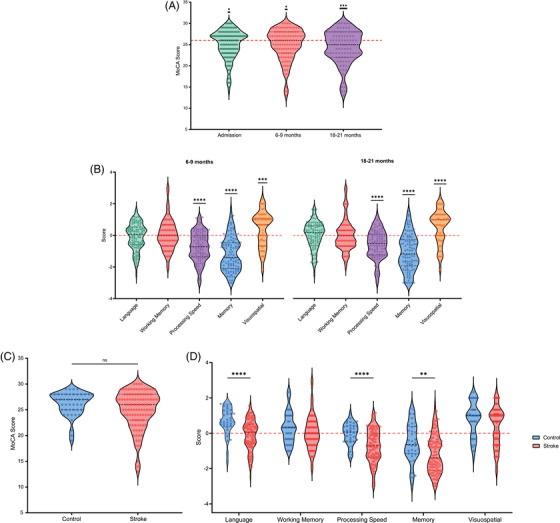
Summary of longitudinal cognitive measures. (A) MoCA scores assessed at admission (*n* = 140), 6–9 months (*n* = 108) and 18–21 months (*n* = 95) post‐ischemic stroke. Scores at each timepoint were compared to the cognitive impairment threshold of 26 (red dashed line) using one‐sample Wilcoxon signed‐rank tests. (B) Domain‐specific Z‐scores at 6–9 months (*n* = 110) and 18–21 months (*n* = 108) were compared to 0 (red dashed line) using one‐sample Wilcoxon signed‐rank tests. (C) MoCA scores were compared between ischemic stroke patients at 6–9 months and non‐stroke control participants using a Mann–Whitney test. (D) Z‐scores for language, working memory, processing speed, memory and visuospatial domains were compared between ischemic stroke patients at 6–9 months and non‐stroke controls using Mann–Whitney tests. * = *p *< 0.05; ** = *p *< 0.01; *** = *p *< 0.001; **** = *p *< 0.0001. MoCA, Montreal Cognitive Assessment.

In the ischemic stroke cohort, MoCA scores were assessed at admission, 6–9 months, and 18–21 months, and were significantly lower than the commonly used threshold for cognitive impairment of 26 at all timepoints (Figure 3A; Supplementary Table S3). Domain‐specific analyses showed significantly poorer performance in memory and processing speed/executive function at both 6–9 and 18–21 months for ischemic stroke patients (Figure 3B; Supplementary Table S4). Cognitive performance in the ischemic stroke cohort at the 6‐ to 9‐month timepoint was then compared with vascular‐risk controls, as control data were only available at one timepoint. MoCA scores were unchanged between controls and stroke patients (Figure 3C). However, stroke participants demonstrated significantly lower domain‐specific performance in language, memory, and processing speed/executive function (Figure 3D; Supplementary Table S5). For subsequent analyses, a binary indicator of global cognitive impairment was used, along with the z‐scores for most affected domains in our ischemic stroke cohort – processing speed/executive function and memory.

### 3.4 Post‐stroke plasma IL‐6 or other inflammatory factors and relationships with 18‐ to 21‐month cognition

All subsequent analyses were restricted to the stroke cohort, as longitudinal biomarker and cognitive data were not available for controls. Plasma IL‐6 concentrations measured at admission were not associated with global cognitive status at 18–21 months (Supplementary Table S6) but were related to poorer processing speed/executive function at this timepoint (Supplementary Table S7). In contrast, IL‐6 at 6–9 months showed stronger associations, relating to worse global cognition, poorer processing speed/executive function and memory at 18–21 months (Supplementary Tables S8–S9). No other inflammatory factors measured at admission or 6–9 months were associated with cognitive outcomes at 18–21 months (Supplementary Tables S10–S11).

**FIGURE 4:**
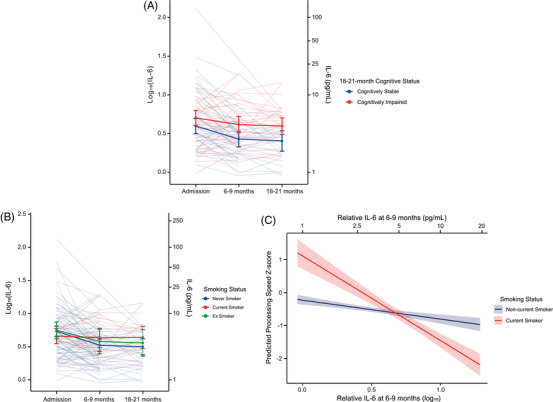
IL‐6 trajectories following ischemic stroke and modification by smoking status. (A) Trajectories of IL‐6 concentrations at admission, 6–9 months and 18–21 months post‐stroke, stratified by cognitive impairment at 18–21 months. Thin lines represent individual unadjusted trajectories; bold lines indicate EMMs for cognitively impaired (red) and cognitively stable (blue) individuals at the 18–21‐month timepoint. (B) Trajectories of IL‐6 concentrations at admission, 6–9 months and 18–21 months post‐stroke, stratified by pre‐stroke smoking history. Thin lines represent individual unadjusted trajectories; bold lines indicate EMMs for current smokers (red), ex‐smokers (green), and never smokers (blue) at the time of stroke. (C) Interaction between relative change in IL‐6 from admission to 6–9 months and smoking status on predicted processing speed at 18–21 months, derived from linear regression models. Lines represent model‐based predictions with 95% confidence intervals for current smokers (red) and non‐current smokers (blue). EMM, estimated marginal mean; IL, interleukin.

Exploratory inspection of longitudinal IL‐6 trajectories showed persistently elevated IL‐6 levels in cognitively impaired individuals, while non‐impaired individuals exhibited a clearer decline, with the greatest separation between these groups seen at 6–9 months post‐stroke (Figure 4A). Accordingly,[Table alz71261-tbl-0002] longitudinal increases in IL‐6 were strongly associated with adverse cognitive outcomes. Doubling of IL‐6 concentrations between admission and 6–9 months was associated with eight‐fold higher odds of cognitive impairment at 18–21 months (OR = 8.16, 95% CI 1.82–47.26; *p *= 0.01) (Table 2). In sensitivity analyses, addition of pre‐stroke mRS attenuated the estimated effect size for the association between doubling of IL‐6 and cognitive impairment, but remained significant (Supplementary Table S12). Further, increases in IL‐6 over this period were also associated with poorer memory performance (β = ‐1.50; 95% CI ‐2.57–0.43; *p *= 0.007) (Table 3), which remained robust after correction for heteroscedasticity. Sensitivity analyses incorporating pre‐stroke mRS score were undertaken for all other models and did not materially alter statistical significance, with only slight attenuation of effect sizes (data not shown).

**TABLE 2 alz71261-tbl-0002:** Relative change in plasma IL‐6 concentration from admission to 6–9 months and association with global cognitive impairment at 18–21 months.

Predictor	OR	95% CI	*p*‐value
IL‐6 at 6–9 months (doubling)	8.16	1.82‚ 47.26	**0.01** ^*^
Age (per year)	0.97	0.91‚ 1.03	0.27
Sex (female vs male)	0.74	0.21‚ 2.52	0.64
NIHSS (per point)	1.00	0.88‚ 1.13	0.97
Diabetes (yes vs no)	0.85	0.23‚ 3.04	0.80
Hypertension (yes vs no)	0.87	0.29‚ 2.61	0.80
IL‐6 at admission (doubling)	0.70	0.15‚ 3.03	0.64
Time to 6‐ to 9‐month follow‐up (days)	0.99	0.97‚ 1.01	0.23

*Note*: ORs with 95% CIs and *p‐*values are shown from a logistic regression model, adjusted for demographic and clinical covariates, as well as IL‐6 concentration at admission. IL‐6 is expressed per doubling.

Abbreviations: CI, confidence interval; IL, interleukin; NIHSS, National Institute of Health Stroke Scale; OR, odds ratio.

* *p *< 0.05.

**TABLE 3:** Relative change in plasma IL‐6 concentration from admission to 6–9 months and associations with domain‐specific z‐scores at 18–21 months.

Domain	β	95% CI	*p*‐value
Processing speed/executive function	−0.69	−1.55, 0.18	0.12
Memory	−1.50	−2.57, ‐0.43	**0.007** ^**^

*Note*: βs with 95% CIs and *p*‐values are shown from linear regression models, adjusted for demographic and clinical covariates, as well as IL‐6 concentration at admission.

Abbreviations: β, beta coefficient; CI, confidence interval; IL, interleukin.

** = *p *< 0.01.

### 3.5 Smoking, IL‐6, and post‐stroke cognitive outcomes

As IL‐6 concentrations at 6–9 months were strongly associated with cognitive outcomes, we next examined whether clinical factors may influence IL‐6 at this timepoint. Among all demographic and clinical variables tested, only smoking status prior to stroke was associated with plasma IL‐6 at 6–9 months (W = 746, *p* = 0.01) (Supplementary Table S13). All individuals who were current smokers at the time of stroke continued smoking at follow‐up.

Longitudinal modelling revealed distinct IL‐6 trajectories when stratified by smoking status. Ex‐ and never‐smokers exhibited increased IL‐6 acutely following ischemic stroke, followed by a marked decline in IL‐6 between admission and 6–9 months (Figure 4B; Supplementary Table S14). In contrast, although current smokers had lower IL‐6 concentrations at admission, they showed little change in IL‐6 over time, resulting in persistently higher levels at both 6–9 months (β = 0.18, 95% CI 0.02–0.35, *p *< 0.05) and 18–21 months (β = 0.21, 95% CI 0.02–0.41, *p *< 0.05).

Given the similar IL‐6 trajectories observed in current smokers and cognitively impaired participants, we next examined whether smoking modified the association between post‐stroke IL‐6 and subsequent cognitive outcomes. Among current smokers, greater increases in IL‐6 between admission and 6–9 months were associated with poorer processing speed/executive function at 18–21 months (β = ‐2.09, 95% CI ‐3.97−‐0.21, *p* < 0.05) (Figure 4C; Supplementary Table S15). No significant associations were observed for memory or global cognition.

### 3.6 Transcriptomic profiling of IL‐6 signaling after ischemic stroke

We next sought to investigate the potential mechanisms underlying the flat trajectory of IL‐6 in current smokers post‐ischemic stroke. Bulk RNA sequencing was performed on PBMCs collected at admission from a subset of participants (*n* = 84), and stratified by smoking status (current smokers, *n* = 21; non‐current smokers, *n* = 63).

Gene set enrichment analysis revealed significant downregulation of TLR signaling in current smokers acutely after ischemic stroke, a key pathway involved in IL‐6 induction (normalized enrichment score [NES] = −1.48, adjusted *p *< 0.05). TLR pathway activity scores, derived using gene set variation analysis, were positively associated with plasma IL‐6 concentrations at admission (β = 5.71, 95% CI 1.01−10.41, *p *< 0.05) (Supplementary Figure S2A; Supplementary Table S16).

Finally, to confirm the expected dynamics of IL‐6 following stroke, a linear mixed‐effects model was fitted. As expected, participants with higher admission IL‐6 (top tercile) showed greater declines between admission and 6–9 months, whereas those with lower admission IL‐6, as observed in smokers, exhibited flatter trajectories (Supplementary Figure S2B; Supplementary Table S17).

### 3.7 Pathways linking socioeconomic status, smoking, IL‐6 and cognition

**FIGURE 5:**
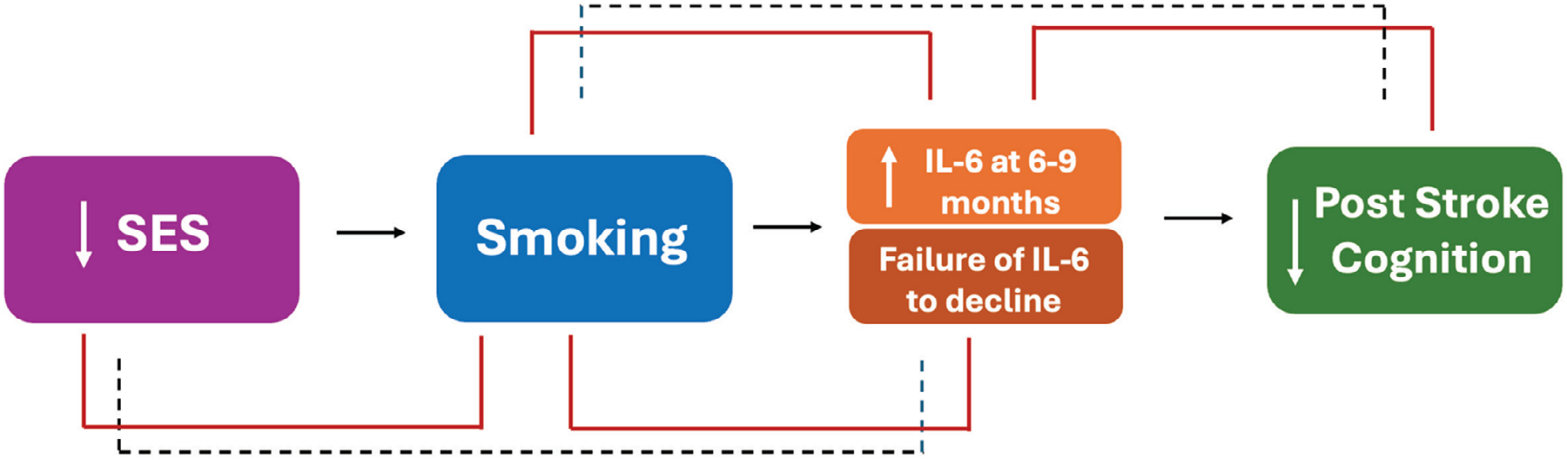
Mediation analysis of the relationship between socioeconomic status, smoking, circulating IL‐6 trajectory and cognition following ischemic stroke. Pathway diagram showing the relationships between low SES, smoking, change in IL‐6 concentration within 6–9 months post‐ischemic stroke, and cognitive outcomes at 18–21 months. Red solid lines indicate significant mediation pathways: smoking mediates the relationship between SES and relative IL‐6 change (*p *< 0.01), and elevated IL‐6 at 6–9 months mediates the relationship between smoking and worse cognitive outcomes (*p *< 0.05). The grey dashed line indicates that the overall indirect effect of SES on cognition via smoking and IL‐6 was not significant in the two‐step mediation analysis. Arrows indicate direction of associations: lower SES (indicated by downward arrow) is associated with smoking, smoking is associated with elevated plasma IL‐6 at 6–9 months following ischemic stroke (indicated by upward arrow), and elevated IL‐6 is associated with worse post‐stroke cognition (indicated by downward arrow). Analysis was adjusted for age, sex, stroke severity (NIHSS), and admission IL‐6 concentration (*n* = 96). IL, interleukin; NIHSS, National Institute of Health Stroke Scale; SES, socioeconomic status.

Given the potential link between smoking and deprivation, we examined whether socioeconomic status influenced plasma IL‐6 concentrations. Higher deprivation was associated with elevated IL‐6 at 6–9 months (Supplementary Table S18), an association that was attenuated after adjustment for smoking status, prompting exploratory mediation analysis.  Mediation analyses in a limited sample suggested that smoking may partially account for the relationship between higher deprivation (Index of Multiple Deprivation) and elevated IL‐6 at 6–9 months (*p *< 0.05, *n* = 96) (Supplementary Table S19). Extending this pathway to cognition, mediation analyses again suggested that relative changes in IL‐6 between admission and 6–9 months mediated the association between smoking and poorer memory and global cognition, with a similar trend observed for processing speed/executive function (Supplementary Tables S20–S22; Figure 5). However, causal direction cannot be inferred.

## 4 DISCUSSION

In the present study, we identified that longitudinal changes in plasma IL‐6 concentrations after ischemic stroke were independently associated with long‐term cognitive outcomes. Specifically, a doubling of IL‐6 concentrations between admission and 6–9 months was associated with eight‐fold increased risk of global cognitive impairment at 18–21 months, as well as worse memory performance. Further, exploratory analyses suggested that smoking may modify the trajectory of plasma IL‐6; current smokers at the time of ischemic stroke showed blunted IL‐6 profiles at admission, which remained persistently flat through to 18–21 months, in contrast to the acute rise then gradual fall patterns observed in ex‐ and never‐smokers. Notably, among current smokers, the association between increasing IL‐6 and poorer processing speed scores at 18–21 months was stronger. Further, transcriptomic analyses revealed downregulation of TLR signaling in these individuals, a key pathway for IL‐6 induction, suggesting a possible smoking‐related vulnerability in post‐stroke inflammatory regulation and cognitive recovery.

Our findings align with prior studies implicating circulating IL‐6 concentrations in poorer post‐stroke functional and cognitive recovery.^9^, ^16^, ^23^, ^24^, ^48^ Specifically, previous work has reported cross‐sectional relationships between circulating IL‐6 acutely (< 4 days) post‐stroke and worse MoCA scores at 3, 18, and 36 months, as well as 18–month IL‐6 measurements with 36‐month MoCA scores.^9^ Our work reinforces these findings, demonstrating the relevance of temporal dynamics of IL‐6, and capturing associations with more extensive cognitive measures. Earlier reports showing associations between admission IL‐6 and later cognition may, in part, capture patients whose IL‐6 remained persistently elevated from admission to 6–9 months, suggesting impaired resolution of inflammation. Overall, these findings indicate that IL‐6 may capture aspects of post‐stroke inflammatory regulation, rather than simply demonstrating inflammation at a given time point.

Following ischemic brain injury, the physiological immune response is typically biphasic: an acute rise in IL‐6 and other mediators mobilizes repair processes, followed by gradual resolution of inflammation as homeostasis is restored.^11^, ^49^ The initial phase clears damaged tissue, activates repair pathways, and recruits regenerative cells, while the resolution phase prevents excess injury and enables recovery.^50^ We propose that patients with persistently flat IL‐6 trajectories may represent those with impaired regulation of post‐stroke inflammatory processes, either failing to mount an adequate pro‐inflammatory response or failing to resolve the initial wave of inflammation. We suggest that both mechanisms could, in turn, be associated with poorer cognitive outcomes. The temporal pattern of our results supports this interpretation, as admission IL‐6 was only weakly associated with cognition compared with IL‐6 levels at 6–9 months.

Transcriptomic analyses revealed downregulation of TLR signaling in current smokers at the time of stroke, a pathway involved in IL‐6 induction. However, these findings were exploratory and in a smaller sub‐cohort, thus require replication in larger cohorts. Importantly, patients with higher admission IL‐6 concentrations subsequently demonstrated greater decline by 6–9 months, suggesting that a robust initial response may in turn facilitate resolution – a process that potentially appears blunted in smokers. Consistent with this interpretation, smoking has been linked to poorer recovery following myocardial infarction,^51^ traumatic brain injury,^52^ impaired immune cell activity^53^, ^54^ and declining cognition.^55^, ^56^

Further exploratory analyses suggested that smoking may partially account for an association between higher socioeconomic deprivation and elevated IL‐6 concentrations. Although in a limited number of individuals, we speculate that socioeconomic deprivation is not a direct determinant of inflammatory responses, but rather a composite marker, capturing cumulative vascular and lifestyle burden that may influence post‐stroke outcomes, of which smoking represents one observable component. Other vascular and behavioral factors are likely to contribute, however, model constraints limited further adjustment.

In addition to IL‐6, eight other inflammatory factors were measured in this study. However, we did not observe similarly robust associations with cognition for other inflammatory markers tested, including CCL3^9^ and MMP‐9,^57^ which have been previously linked to PSCD. Discrepancies may be explained by differences in cohort size, sampling timepoints, stroke severity, or cognitive assessment methods and timing. Notably, both our study (median sampling time of 45 hours post‐stroke) and the Nor‐COAST study (median of 4 days)^9^ did not observe significant associations with MMP‐9, whereas studies sampling much earlier (< 24 hours) did.^57^ This pattern highlights that understanding the temporal dynamics of these plasma factors is crucial to uncovering their cognitive relationships; some inflammatory mediators, such as MMP‐9, may be acutely responsive to ischemic injury, while others, including IL‐1Ra, may show more delayed or prolonged kinetics.^58^

The present study has several notable methodological strengths. The longitudinal design with serial blood biomarker measurements over 18 months enabled us to capture immune system dynamics over time, rather than relying on single timepoints. Our study employed a comprehensive neuropsychological battery, enabling the detection of more subtle or domain‐specific deficits, rather than relying solely on screening tools such as the Mini‐Mental State Examination or MoCA. Incorporation of socioeconomic status and smoking status allowed us to examine important modifiers of immune function. The integration of transcriptomic analyses further strengthened the biological plausibility of our findings by identifying possible mechanisms underlying the observed associations.

Conversely, several limitations should also be acknowledged. The sample size (*n* = 190) was modest, with complete biomarker data at admission and 6–9 months, and cognitive data at 18–21 months, available for only 77 ischemic stroke participants. Hence, this may have reduced the power to detect more subtle analyte‐cognition relationships, particularly for analytes other than IL‐6. Further, measurements of biomarkers were only available for 11 non‐stroke controls for analytes aside from IL‐6, which may have limited our ability to assess stroke‐associated changes in these factors. The modest sample size of the study was in part due to patient attrition as well as funding limitations. Additionally, loss to follow‐up likely introduced attrition bias. Although patients were excluded if they had a formal dementia diagnosis, dementia and cognitive decline may be underdiagnosed prior to stroke, and unmeasured premorbid cognitive impairment cannot be fully excluded. Additionally, cognitive performance was assessed at 6–9 months post‐stroke, whereas non‐stroke controls were assessed at a single time point, limiting direct comparability and precluding assessment of control cognitive trajectories. Plasma amyloid and tau concentrations were not measured in the present study, however, would be of interest. The observational design precludes causal inference, and pre‐clinical models are required to establish mechanistic links between the immune dynamics and cognition proposed here. Aside from smoking status, unmeasured interval inflammatory exposures may have contributed to variability in biomarker levels, including post‐stroke medication usage, recurrent stroke and post‐stroke infection, which were not collected prospectively during study follow‐up. Further, we were unable to explore longer‐term cognitive trajectories post‐stroke. While cognitive impairment can be evident within the first‐year post‐stroke, longer‐term follow‐up may be informative for characterizing cognitive trajectories rather than cross‐sectional deficit.^59^ Ongoing cognitive assessments at 30–33 months may provide further insight into whether plasma IL‐6 is associated with longer‐term cognitive trajectories, but sample size and participant attrition will increase uncertainty in the estimates. The small number of current smokers (*n* = 21) in transcriptomic analysis should be considered exploratory and requires validation in larger cohorts. Socioeconomic status was approximated using postcode‐derived indices, which may not fully capture individual circumstances. Finally, the single‐center design and limited demographic diversity of our cohort may limit generalizability.

Nevertheless, these findings may have translational relevance. Longitudinal IL‐6 monitoring post‐stroke, particularly in the first 6–9 months, could aid prognostication and risk stratification, helping to identify patients at the highest risk of cognitive decline who may benefit from closer monitoring or targeted cognitive training.^60^, ^61^ If a causal role for IL‐6 in PSCD could be established, anti‐IL‐6 therapies such as tocilizumab warrant exploration as potential strategies to mitigate post‐stroke cognitive impairment within this time window.^62^, ^63^ Further investigation would be required to determine the timing of when these therapies should be administered; it is likely that IL‐6 should not be blocked too soon following ischemic stroke, as the acute inflammatory surge may play a necessary role in recovery. Instead, targeting patients who fail to resolve IL‐6 concentrations in the subacute phase may represent a more effective and safer therapeutic strategy, although further work would also have to define a threshold concentration of IL‐6 at which to consider treatment.

Future work should aim to externally validate these findings in a larger dataset with longitudinal IL‐6 measurements to better characterize immune trajectories after stroke. Mendelian randomization studies could help determine whether IL‐6 signaling plays a causal role in PSCD. Additionally, analyses of existing datasets, from pre‐clinical studies to clinical trials, could investigate whether anti‐IL‐6 therapies, such as tocilizumab, have a beneficial effect on cognitive outcomes.

Overall, our findings suggest that impaired resolution of IL‐6–driven inflammation, rather than the initial inflammatory magnitude alone, is associated with poorer long‐term cognitive outcomes after ischemic stroke.  Notably, a doubling of IL‐6 concentrations within the first 6–9 months post‐stroke was associated with an approximately eight‐fold increased risk of subsequent cognitive impairment. Identifying the importance of IL‐6 dynamics opens new avenues for risk stratification and targeted intervention. Specifically, the first 6–9 months following ischemic stroke may represent a critical recovery window during which failure to downregulate inflammation leads to long‐term cognitive consequences.

## AUTHOR CONTRIBUTIONS

M.S.B., J.R.G., S.M.A. and C.J.S. designed the clinical study, provided supervision and contributed to interpretation of data and manuscript preparation. N.S.C. conducted laboratory experiments, performed data analyses and wrote the first manuscript draft. H.R.D. performed laboratory experiments, data analysis and manuscript preparation. S.Y.W. conducted sample processing and coordinated laboratory procedures. T.O.W. assisted with laboratory experiments. E.K. provided guidance on statistical analyses. E.J. managed the clinical study and data collection, and L.C. conducted cognitive assessments, data collection and assisted with data organisation. L.D. is the study neuropsychologist and was responsible for cognitive assessments.

## CONFLICT OF INTEREST STATEMENT

The authors declare no conflicts of interest. Author disclosures are available in the supporting information.

## CONSENT STATEMENT

All participants in the present work consent to the use of their data and clinical information.

## Supporting information



Supporting Information

Supporting Information

Supporting Information

Supporting Information
