# Supplementary material for: Longitudinal plasma interleukin‐6 and post‐stroke cognitive outcomes: The Stroke‐IMPaCT study
Source: Alzheimers Dement. 2026 Mar 10;22(3):e71261. doi: 10.1002/alz.71261 (PMC12973141; doi:10.1002/alz.71261)
Supplement: Supplementary file 1 — Supporting Information [file ALZ-22-e71261-s003.docx]

**Supplementary Tables**

**Supplementary Table 1 – Plasma inflammatory factor concentrations in non-stroke, vascular-risk controls and ischaemic stroke patients.** Data presented as medians [IQRs]. *n=45 for IL-6 concentrations from non-stroke controls.

|  | **Plasma concentration (median, [IQR])** | | | |
| --- | --- | --- | --- | --- |
| **Inflammatory Factor** | **Non-stroke controls**  **(n=11)** | **Stroke, admission**  **(n=178)** | **Stroke, 6-9 months**  **(n=97)** | **Stroke, 18-21 months**  **(n=53)** |
| CCL2  *(pg/mL)* | 208 [198, 311] | 235 [181, 290] | 234 [195, 272] | 243 [197, 286] |
| CCL3  *(pg/mL)* | 30.6 [20.0, 35.2] | 31.2 [24.2, 41.8] | 27.2 [23.3, 35.8] | 28.5 [23.1, 36.0] |
| CD163  *(ng/mL)* | 501.3 [373.7, 594.2] | 431.2 [341.5, 626.2] | 476.5 [375.6, 638.0] | 447.7 [380.3, 641.9] |
| CRP  *(mg/L)* | 0.8 [0.7, 2.2] | 3.2 [1.4, 7.4] | 1.2 [0.6, 2.9] | 0.9 [0.5, 1.7] |
| IL-1Ra  *(pg/mL)* | 342 [253, 449] | 394 [292, 568] | 302 [236, 438] | 348 [252, 456] |
| IL-6  *(pg/mL)* | 3.0 [1.8, 4.2] * | 4.6 [2.9, 8.6] | 3.3 [2.2, 5.6] | 3.2 [2.0, 5.3] |
| IL-8  *(pg/mL)* | 7.8 [7.6, 10.0] | 7.8 [5.8, 10.3] | 7.9 [6.0, 10.3] | 8.4 [6.6, 10.5] |
| IL-18  *(pg/mL)* | 202 [153, 325] | 198 [157, 253] | 223 [186, 270] | 213 [169, 248] |
| MMP-9  *(ng/mL)* | 104.9 [73.2, 236.5] | 143.3 [86.2, 220.5] | 98.1 [60.8, 150.3] | 101.3 [70.0, 213.9] |

**Supplementary Table 2 – Linear mixed-effects model of longitudinal plasma IL-6 trajectories following ischaemic stroke.** Beta coefficients (β) with 95% confidence intervals (CI) and *P* values are shown from a linear-mixed effect model, adjusted for demographic and clinical covariates. ** = *P*<0.01; *** = *P* < 0.001; **** = *P*<0.0001.

| **Predictor** | **β** | **95% CI** | **P value** |
| --- | --- | --- | --- |
| 6-9-month visit | -0.16 | -0.23, -0.10 | **3.9e^-6^ ****** |
| 18-21-month visit | -0.17 | -0.25, -0.08 | **0.0001 ***** |
| Age (per year) | 0.01 | 0.002, 0.01 | **0.006 **** |
| Sex (female vs male) | -0.03 | -0.13, 0.07 | 0.61 |
| NIHSS (per point) | 0.02 | 0.01, 0.03 | **3.4e^-5^ ****** |
| Diabetes (yes vs no) | 0.001 | -0.10, 0.11 | 0.97 |
| Hypertension (yes vs no) | 0.07 | -0.02, 0.16 | 0.13 |

**Supplementary Table 3 – Summary of ischaemic stroke patient MoCA scores at admission, 6-9 and 18-21 months.** One-sample Wilcoxon signed-rank tests were used to compare MoCA scores to 26, the cognitive impairment threshold. Adjustments for multiple comparisons were made with the FDR method. * = *P* < 0.05; ** = *P* < 0.01; *** = *P*<0.001.

| **Timepoint** | **P value** | **FDR-adjusted**  **P value** |
| --- | --- | --- |
| Admission | **0.01 *** | **0.02 *** |
| 6-9 months | **0.04 *** | **0.04 *** |
| 18-21 months | **0.0004 ***** | **0.001 **** |

**Supplementary Table 4 – Summary of ischaemic stroke patient cognitive domain Z-scores at 6-9 and 18-21 months.** One-sample Wilcoxon signed-rank tests were used to compare Z-scores to 0. Adjustments for multiple comparisons were made with the FDR method. *** = *P*<0.001; **** = *P*<0.0001.

| Timepoint | Domain | *P* value | FDR-adjusted  *P* value |
| --- | --- | --- | --- |
| 6-9 months | Memory | **1.4e^-14^ ****** | **2.0e^-13^ ****** |
|  | Processing Speed/executive function | **3.1e^-11^ ****** | **1.5e^-10^ ****** |
|  | Visuospatial | **0.0004 ***** | **0.0006 ***** |
|  | Language | 0.57 | 0.67 |
|  | Working Memory | 0.95 | 0.95 |
| 18-21 months | Memory | **1.7e^-11^ ****** | **1.2e^-10^ ****** |
|  | Processing Speed/executive function | **1.8e^-7^ ****** | **5.1e^-7^ ****** |
|  | Visuospatial | **1.1e^-5^ ****** | **2.2e^-5^ ****** |
|  | Language | 0.50 | 0.63 |
|  | Working Memory | 0.62 | 0.67 |

| Domain | *P* value | FDR-adjusted  *P* value |
| --- | --- | --- |
| Memory | **0.003 **** | **0.005 **** |
| Processing speed/executive function | **3.3e^-5^ ****** | **8.1e^-5^ ****** |
| Visuospatial | 0.09 | 0.12 |
| Language | **3.2e^-6^ ****** | **1.6e^-5^ ****** |
| Working memory | 0.12 | 0.12 |

**Supplementary Table 5 – Summary of ischaemic stroke patient cognitive domain Z-scores at 6-9 months versus non-stroke controls.** Mann Whitney tests were used to compare Z-scores between the two groups. Adjustments for multiple comparisons were made with the FDR method. ** = *P*<0.01; **** = *P*<0.0001.

**Supplementary Table 6 – Plasma IL-6 concentration at admission and association with global cognitive impairment at 18-21 months.** Odds ratios (OR) with 95% confidence intervals (CI) and *P* values are shown from a logistic regression model, adjusted for demographic and clinical covariates. IL-6 is expressed per doubling.

| **Predictor** | **OR** | **95% CI** | **P value** |
| --- | --- | --- | --- |
| IL-6 at admission (doubling) | 2.55 | 0.86‚ 8.55 | 0.11 |
| Age (per year) | 0.97 | 0.92‚ 1.02 | 0.22 |
| Sex (female vs male) | 1.07 | 0.35, 3.27 | 0.91 |
| NIHSS (per point) | 0.97 | 0.86‚ 1.09 | 0.64 |
| Diabetes (yes vs no) | 2 | 0.7, 6.0 | 0.20 |
| Hypertension (yes vs no) | 0.63 | 0.24‚ 1.62 | 0.35 |
| Time to blood draw post-stroke (hours) | 1.03 | 1, 1.06 | 0.06 |

**Supplementary Table 7 – Plasma IL-6 concentration at admission and associations with domain-specific z-scores at 18-21 months.** Beta coefficients (β) with 95% confidence intervals (CI) and *P* values are shown from linear regression models, adjusted for demographic and clinical covariates. ** = *P*<0.01.

| **Domain** | **β** | **95% CI** | **P value** |
| --- | --- | --- | --- |
| Processing speed/executive function | -0.94 | -1.54, -0.35 | **0.002 **** |
| Memory | -0.47 | -1.32, 0.37 | 0.27 |

**Supplementary Table 8 – Plasma IL-6 concentration at 6-9 months and association with global cognitive impairment at 18-21 months.** Odds ratios (OR) with 95% confidence intervals (CI) and *P* values are shown from a logistic regression model, adjusted for demographic and clinical covariates. IL-6 is expressed per doubling. ** = *P*<0.01.

| **Predictor** | **OR** | **95% CI** | **P value** |
| --- | --- | --- | --- |
| IL-6 at 6-9 months (doubling) | 6.51 | 1.79, 29.12 | **0.01 **** |
| Age (per year) | 0.96 | 0.91, 1.02 | 0.18 |
| Sex (female vs male) | 0.88 | 0.27, 2.84 | 0.83 |
| NIHSS (per point) | 1.0 | 0.88, 1.12 | 0.96 |
| Diabetes (yes vs no) | 0.95 | 0.28, 3.14 | 0.93 |
| Hypertension (yes vs no) | 0.71 | 0.25, 1.96 | 0.51 |
| Time to 6-9-month follow-up (days) | 0.99 | 0.97, 1.01 | 0.20 |

**Supplementary Table 9 – Plasma IL-6 concentration at 6-9 months and associations with domain-specific z-scores at 18-21 months.** Beta coefficients (β) with 95% confidence intervals (CI) and *P* values are shown from linear regression models, adjusted for demographic and clinical covariates. * = *P*<0.05; *** = *P*<0.001.

| **Domain** | **β** | **95% CI** | **P value** |
| --- | --- | --- | --- |
| Processing speed/executive function | -0.84 | -1.58, -0.09 | **0.03 *** |
| Memory | -1.58 | -2.48, -0.68 | **0.001 ***** |

**Supplementary Table 10 – Plasma inflammatory factor concentrations at admission and 6-9 months and associations with global cognitive impairment at 18-21 months.** Odds ratios (OR) with 95% confidence intervals (CI) and *P* values are shown from logistic regression models, adjusted for demographic and clinical covariates. Inflammatory factors are expressed per doubling.

| **Inflammatory Factor** | **Timepoint** | **OR** | **95% CI** | ***P* value** |
| --- | --- | --- | --- | --- |
| CCL2 | Admission | 2.77 | 0.37, 24.48 | 0.33 |
|  | 6-9 months | 0.78 | 0.05, 12.58 | 0.86 |
| CCL3 | Admission | 2.94 | 0.79, 17.49 | 0.17 |
|  | 6-9 months | 3.27 | 0.73, 21.35 | 0.16 |
| CD163 | Admission | 0.72 | 0.10, 5.24 | 0.74 |
|  | 6-9 months | 0.51 | 0.08, 3.23 | 0.48 |
| CRP | Admission | 1.57 | 0.87, 2.95 | 0.14 |
|  | 6-9 months | 1.26 | 0.58, 2.78 | 0.56 |
| IL-1Ra | Admission | 3.72 | 0.91, 19.17 | 0.09 |
|  | 6-9 months | 1.07 | 0.23, 5.00 | 0.93 |
| IL-8 | Admission | 0.36 | 0.05, 2.55 | 0.31 |
|  | 6-9 months | 0.11 | 0.01, 1.06 | 0.06 |
| IL-18 | Admission | 1.30 | 0.11, 14.75 | 0.83 |
|  | 6-9 months | 0.46 | 0.04, 5.23 | 0.53 |
| MMP-9 | Admission | 1.16 | 0.39, 3.50 | 0.79 |
|  | 6-9 months | 0.96 | 0.31, 3.00 | 0.95 |

**Supplementary Table 11 – Plasma inflammatory factor concentrations at admission and 6-9 months and associations with domain-specific z-scores at 18-21 months.** Beta coefficients (β) with 95% confidence intervals (CI) and *P* values are shown from linear regression models, adjusted for demographic and clinical covariates.

| **Inflammatory Factor** | **Timepoint** | **Processing Speed/Executive Function** | | | **Memory Factor** | | |
| --- | --- | --- | --- | --- | --- | --- | --- |
|  |  | **β** | **95% CI** | ***P* value** | **β** | **95% CI** | ***P* value** |
| CCL2 | Admission | -0.54 | -1.77, 0.69 | 0.39 | -0.80 | -2.45, 0.84 | 0.33 |
|  | 6-9 months | -0.44 | -2.28, 1.41 | 0.64 | -1.29 | -3.60, 1.02 | 0.27 |
| CCL3 | Admission | -0.35 | -1.02, 0.32 | 0.30 | -0.46 | -1.68, 0.75 | 0.45 |
|  | 6-9 months | -0.45 | -1.37, 0.47 | 0.33 | -1.01 | -2.16, 0.15 | 0.09 |
| CD163 | Admission | -0.06 | -1.26, 1.15 | 0.93 | 0.49 | -1.12, 2.11 | 0.55 |
|  | 6-9 months | 0.02 | -1.21, 1.25 | 0.97 | 0.76 | -0.79, 2.31 | 0.33 |
| CRP | Admission | -0.30 | -0.65, 0.04 | 0.09 | -0.03 | -0.51, 0.46 | 0.92 |
|  | 6-9 months | -0.24 | -0.75, 0.27 | 0.35 | -0.19 | -0.84, 0.46 | 0.56 |
| IL-1Ra | Admission | -0.12 | -0.92, 0.68 | 0.77 | -0.51 | -1.57, 0.56 | 0.35 |
|  | 6-9 months | -0.09 | -1.11, 0.92 | 0.85 | -0.54 | -1.83, 0.75 | 0.41 |
| IL-8 | Admission | 0.01 | -1.18, 1.19 | 0.99 | 0.25 | -1.34, 1.83 | 0.76 |
|  | 6-9 months | 1.05 | -0.40, 2.51 | 0.15 | 0.23 | -1.64, 2.10 | 0.81 |
| IL-18 | Admission | 0.56 | -0.87, 2.00 | 0.44 | 0.98 | -0.96, 2.91 | 0.32 |
|  | 6-9 months | 0.80 | -0.82, 2.42 | 0.33 | 1.56 | -0.47, 3.60 | 0.13 |
| MMP-9 | Admission | -0.02 | -0.68, 0.64 | 0.95 | -0.17 | -1.05, 0.72 | 0.71 |
|  | 6-9 months | -0.11 | -0.86, 0.65 | 0.77 | -0.51 | -1.46, 0.43 | 0.28 |

**Supplementary Table 12 – Sensitivity analysis exploring whether pre-stroke modified Rankin Scale (mRS) score affects association between relative change in plasma IL-6 concentration from admission to 6-9 months and association with global cognitive impairment at 18-21 months.** Odds ratios (OR) with 95% confidence intervals (CI) and *P* values are shown from a logistic regression model, adjusted for demographic and clinical covariates as prior, as well as pre-stroke mRS score. IL-6 is expressed per doubling. **P*<0.05.

| **Predictor** | **OR** | **95% CI** | **P value** |
| --- | --- | --- | --- |
| IL-6 at 6-9 months (doubling) | 2.47 | 1.27, 5.36 | **0.01 **** |
| Age (per year) | 0.96 | 0.90, 1.02 | 0.22 |
| Sex (female vs male) | 1.44 | 0.41, 5.32 | 0.57 |
| NIHSS (per point) | 1.01 | 0.89, 1.15 | 0.89 |
| Diabetes (yes vs no) | 0.77 | 0.20, 2.80 | 0.69 |
| Hypertension (yes vs no) | 0.87 | 0.28, 2.68 | 0.81 |
| IL-6 at admission (doubling) | 0.76 | 0.29, 1.94 | 0.57 |
| Time to 6-9-month follow-up (days) | 0.99 | 0.97, 1.01 | 0.25 |
| Pre-stroke mRS score | 1.92 | 0.7, 6.17 | 0.22 |

**Supplementary Table 13 – Plasma IL-6 concentration at 6-9 months and relationships with clinical features.** Group differences were assessed using Mann–Whitney tests. Features in the analysis included demographic information, stroke-related variables, cardiovascular risk factors and pre-stroke medication usage (drug classes). Sample size was n = 97, except for lesion location (n = 90) and frequent alcohol consumption (n = 94). * = *P*<0.05.

| Variable | *P* value |
| --- | --- |
| Smoking Status | **0.01*** |
| Calcium Channel Blockers | 0.06 |
| Atrial Fibrillation | 0.08 |
| Angiotensin Receptor Blockers | 0.09 |
| Antidepressants | 0.10 |
| Beta Blockers | 0.12 |
| Antidiabetics | 0.18 |
| Other Antihypertensives | 0.19 |
| Diabetes | 0.22 |
| Hyperlipidaemia | 0.22 |
| Angiotensin-Converting Enzyme Inhibitors | 0.26 |
| Infarct Location | 0.27 |
| Antiplatelets | 0.28 |
| Hypertension | 0.35 |
| Previous Stroke | 0.38 |
| Intravenous Thrombolysis | 0.54 |
| Alcohol consumption more than once week | 0.72 |
| Sex | 0.78 |
| Heart Disease | 0.92 |
| Antihyperlipidemics | 0.96 |

**Supplementary Table 14 – Interaction between plasma IL-6 and smoking status over time following ischaemic stroke.** Beta coefficients (β) with 95% confidence intervals (CI) and *P* values are shown from a linear mixed-effects model, adjusted for demographic and clinical covariates. An interaction term for the effect of smoking on IL-6 concentration was included. The model included a random intercept for each participant. * =*P*<0.05; *** = *P*<0.001; **** = *P*<0.0001.

| **Predictor** | **β** | **95% CI** | **P value** |
| --- | --- | --- | --- |
| 6-9-month visit | -0.21 | -0.29, -0.13 | **2.3e^-6^ ****** |
| 18-21-month visit | -0.23 | -0.35, -0.12 | **0.0001 ***** |
| Smoking Status (Current Smoker) | -0.07 | -0.20, 0.05 | 0.27 |
| Smoking Status (Ex Smoker) | 0.02 | -0.12, 0.15 | 0.81 |
| Age (per year) | 0.01 | 0.002, 0.01 | **0.01 *** |
| Sex (female vs male) | -0.02 | -0.12, 0.08 | 0.72 |
| NIHSS (per point) | 0.02 | 0.01, 0.03 | **0.0001 ***** |
| Diabetes (yes vs no) | -0.01 | -0.11, 0.10 | 0.91 |
| Hypertension (yes vs no) | 0.08 | -0.01, 0.17 | 0.11 |
| 6-9-month visit : Current Smoker | 0.18 | 0.02, 0.35 | **0.03 *** |
| 18-21-month visit : Current Smoker | 0.21 | 0.02, 0.41 | **0.04 *** |
| 6-9-month visit : Ex Smoker | 0.04 | -0.16, 0.24 | 0.69 |
| 18-21-month visit : Ex Smoker | 0.04 | -0.18, 0.26 | 0.73 |

**Supplementary Table 15 – Interaction between relative change in plasma IL-6 concentration from admission to 6-9 months and smoking status on 18-21-month processing speed/executive function.** Beta coefficients (β) with 95% confidence intervals (CI) and *P* values are shown from linear regression model, adjusted for demographic and clinical covariates, as well as IL-6 at admission. An interaction term for the effect of smoking on IL-6 concentration was included. * = *P*<0.05.

| **Predictor** | **β** | **95% CI** | **P value** |
| --- | --- | --- | --- |
| IL-6 at 6-9 months | -0.52 | -1.47, 0.43 | 0.28 |
| Smoking Status (Current Smoker) | 1.54 | 0.14, 2.93 | **0.03 *** |
| Age (per year) | 0.01 | -0.01, 0.04 | 0.30 |
| Sex (female versus male) | 0.003 | -0.51, 0.52 | 0.99 |
| NIHSS (per point) | -0.05 | -0.11, 0.001 | 0.05 |
| Diabetes (yes vs no) | -0.12 | -0.65, 0.41 | 0.65 |
| Hypertension (yes vs no) | -0.12 | -0.58, 0.33 | 0.59 |
| Time to 6–9-month follow-up (days) | 0.0002 | -0.01, 0.01 | 0.54 |
| IL-6 at admission | -0.04 | -0.94, 0.87 | 0.94 |
| IL-6 at 6-9 months : Current Smoker | -2.09 | -3.97, -0.21 | **0.03 *** |

**Supplementary Table 16 – Association between Toll-like receptor (TLR) score and IL-6 concentration at admission.** Beta coefficients (β) with 95% confidence intervals (CI) and *P* values are shown from a linear regression model, adjusted for demographic and clinical covariates. * = *P*<0.05.

| **Predictor** | **β** | **95% CI** | **P value** |
| --- | --- | --- | --- |
| TLR Score | 5.71 | 1.01, 10.41 | **0.02 *** |
| Age (per year) | 0.06 | -0.15, 0.26 | 0.57 |
| Sex (female vs male) | 0.03 | -0.38, 0.44 | 0.89 |
| NIHSS (per point) | 0.29 | 0.09, 0.5 | **0.01 *** |
| Diabetes (yes vs no) | -0.2 | -0.74, 0.33 | 0.45 |
| Hypertension (yes vs no) | 0.24 | -0.16, 0.63 | 0.24 |

**Supplementary Table 17 – Linear mixed-effects model examining how admission IL-6 concentration predicts longitudinal trajectory.** Beta coefficients (β) with 95% confidence intervals (CI) shown from the model, adjusted for demographic and clinical covariates. IL-6 concentrations at admission were divided into tertiles (low, medium, and high).

| **Admission Tercile** | **Timepoint** | **β** | **95% CI** |
| --- | --- | --- | --- |
| Low | Admission | 0.31 | 0.24, 0.39 |
| Low | 6-9 months | 0.32 | 0.23, 0.42 |
| Low | 18-21 months | 0.31 | 0.20, 0.42 |
| Medium | Admission | 0.65 | 0.58, 0.72 |
| Medium | 6-9 months | 0.57 | 0.48, 0.66 |
| Medium | 18-21 months | 0.61 | 0.50, 0.72 |
| High | Admission | 1.13 | 1.06, 1.20 |
| High | 6-9 months | 0.69 | 0.60, 0.77 |
| High | 18-21 months | 0.60 | 0.46, 0.74 |

**Supplementary Table 18 – Plasma IL-6 concentration at 6-9 months and associations with deprivation domains.** Beta coefficients (β) with 95% confidence intervals (CI) and *P* values are shown from linear regression models, adjusted for demographic and clinical covariates. ** = *P*<0.01; * = *P*<0.05.

| **Deprivation Domain** | **β** | **95% CI** | **P value** |
| --- | --- | --- | --- |
| Education | -1.12 | -1.91, -0.34 | **0.01 **** |
| Employment | -0.87 | -1.65, -0.08 | **0.03 *** |
| Income | -1.07 | -1.95, -0.20 | **0.02 *** |
| Health | -0.69 | -1.37, -0.01 | **0.05 *** |
| Crime | -0.48 | -1.36, 0.41 | 0.29 |
| Housing | 0.41 | -0.36, 1.17 | 0.30 |
| Environment | -0.15 | -0.90, 0.61 | 0.71 |

**Supplementary Table 19 – Pathway from Index of Multiple Deprivation to relative change in plasma IL-6 concentration from admission to 6-9 months through smoking status.** Beta coefficients (β) with 95% confidence intervals (CI) and *P* values are shown from mediation analysis, adjusted for demographic and clinical covariates, as well as admission IL-6. Smoking status was defined as current smoker versus non-current smoker. ** = *P*<0.01.

| **Effect** | **β** | **95% CI** | **P value** |
| --- | --- | --- | --- |
| ACME (indirect) | -0.02 | -0.05, -0.01 | **0.006 **** |
| ADE (direct) | -0.02 | -0.06, 0.03 | 0.55 |
| Total Effect | -0.04 | -0.09, 0.01 | 0.09 |
| Prop. Mediated | 0.62 | -2.64, 5.48 | 0.09 |

**Supplementary Table 20 – Pathway from smoking status to 18-21-month memory performance through relative change in plasma IL-6 concentrations from admission to 6-9 months.** Beta coefficients (β) with 95% confidence intervals (CI) and *P* values are shown from mediation analysis, adjusted for demographic and clinical covariates, as well as admission IL-6. * = *P*<0.05.

| **Effect** | **β** | **95% CI** | **P value** |
| --- | --- | --- | --- |
| ACME (indirect) | 0.16 | 0.02, 0.32 | **0.03 *** |
| ADE (direct) | 0.05 | -0.26, 0.40 | 0.73 |
| Total Effect | 0.21 | -0.09, 0.53 | 0.17 |
| Prop. Mediated | 0.78 | -3.55, 5.70 | 0.16 |

**Supplementary Table 21 – Pathway from smoking status to 18–21-month processing speed/executive function through relative change in plasma IL-6 concentrations from admission to 6-9 months.** Beta coefficients (β) with 95% confidence intervals (CI) and *P* values are shown from mediation analysis, adjusted for demographic and clinical covariates, as well as admission IL-6.

| **Effect** | **β** | **95% CI** | **P value** |
| --- | --- | --- | --- |
| ACME (indirect) | -0.23 | -0.50, 0.02 | 0.08 |
| ADE (direct) | 0.21 | -0.46, 0.72 | 0.51 |
| Total Effect | -0.02 | -0.66, 0.53 | 0.93 |
| Prop. Mediated | 12.75 | -9.53, 11.16 | 0.93 |

**Supplementary Table 22 – Pathway from smoking status to global cognitive impairment at 18-21 months through relative change in plasma IL-6 concentrations from admission to 6-9 months.** Beta coefficients (β) with 95% confidence intervals (CI) and *P* values are shown from mediation analysis, adjusted for demographic and clinical covariates, as well as admission IL-6. * = *P*<0.05.

| **Effect** | **β** | **95% CI** | **P value** |
| --- | --- | --- | --- |
| ACME (indirect) | 0.10 | 0.02, 0.32 | **0.03 *** |
| ADE (direct) | 0.05 | -0.26, 0.4 | 0.73 |
| Total Effect | 0.21 | -0.09, 0.54 | 0.17 |
| Prop. Mediated | 0.78 | -3.55, 5.70 | 0.19 |
