# Supplementary material for: Longitudinal plasma interleukin‐6 and post‐stroke cognitive outcomes: The Stroke‐IMPaCT study
Source: Alzheimers Dement. 2026 Mar 10;22(3):e71261. doi: 10.1002/alz.71261 (PMC12973141; doi:10.1002/alz.71261)
Supplement: Supplementary file 2 — Supporting Information [file ALZ-22-e71261-s001.pdf]

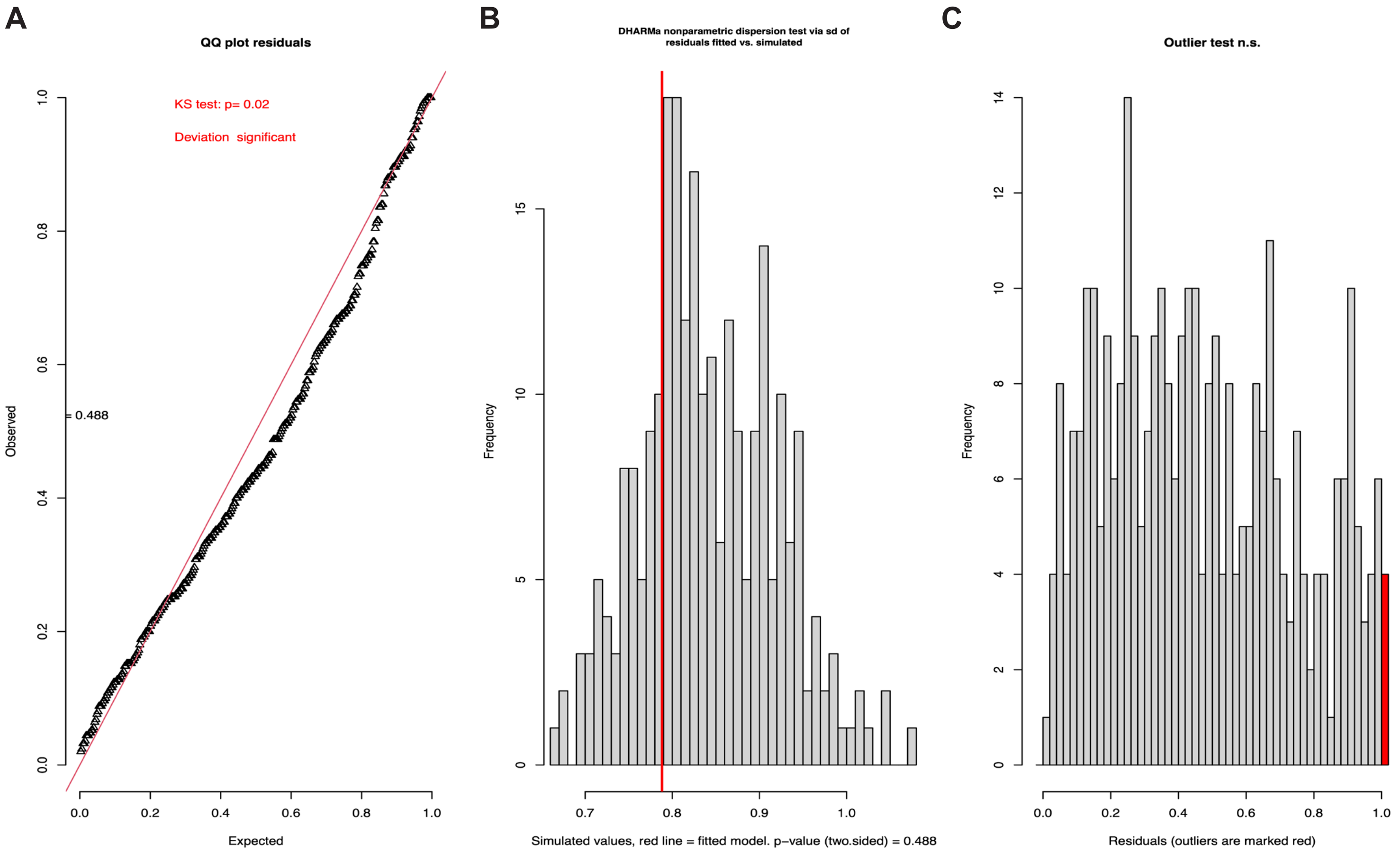

Supplementary Figure 1 – Residual diagnostics for the linear mixed-effects model of post-ischaemic stroke IL-6 trajectories. Plots show A) quantile–quantile (QQ) plot of scaled residuals (Kolmogorov–Smirnov test, *P*=0.02), B) residual dispersion (*P*=0.49), and C) outlier frequency (*P*=0.34). Model diagnostics indicated low multicollinearity (all VIF<2) and moderate explanatory power (marginal R^2 = 0.16; conditional R^2 = 0.57). While the Kolmogornov-Smirnov test indicated a mild deviation from normality, visual inspection of residual plots suggested no substantial departures. No evidence of overdispersion or excess outliers was observed.
