# Supplementary material for: Longitudinal plasma interleukin‐6 and post‐stroke cognitive outcomes: The Stroke‐IMPaCT study
Source: Alzheimers Dement. 2026 Mar 10;22(3):e71261. doi: 10.1002/alz.71261 (PMC12973141; doi:10.1002/alz.71261)
Supplement: Supplementary file 3 — Supporting Information [file ALZ-22-e71261-s002.pdf]

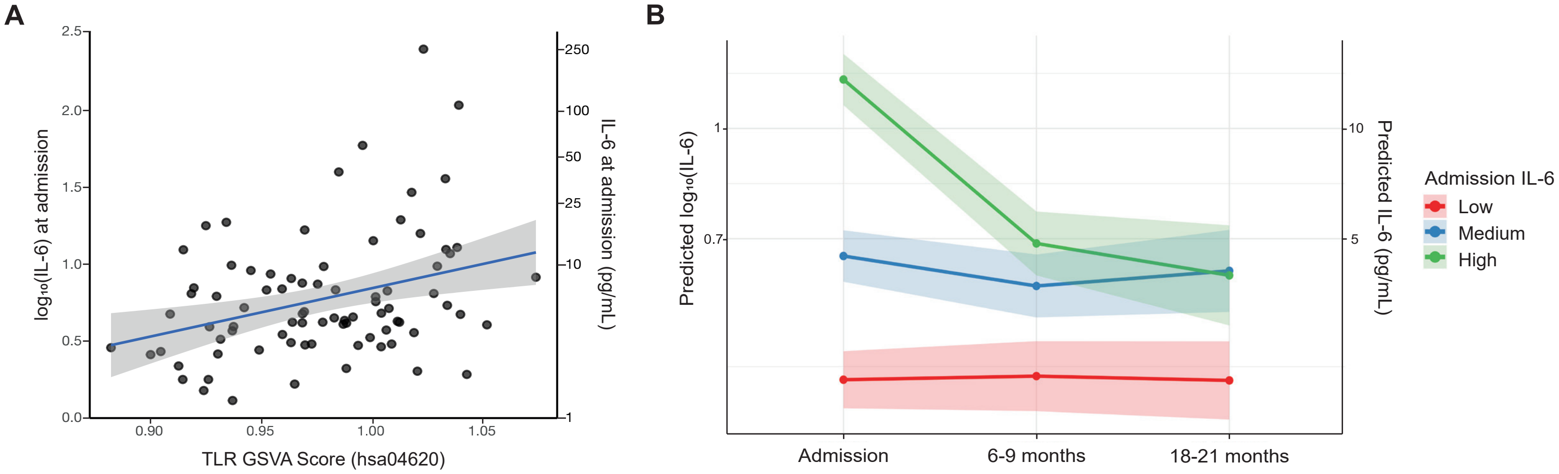

Supplementary Figure 2 – IL-6 association with Toll-like receptor (TLR) enrichment and predicted longitudinal trajectories post-ischaemic stroke. A) Association between Toll-like receptor (TLR) signalling pathway scores and IL-6 at admission. Scatter plot showing sample-level TLR pathway enrichment scores plotted against IL-6 concentrations at admission. The fitted regression line represents the association estimated from a multivariable linear regression model. The shaded area denotes the 95% confidence interval. B) Predicted IL-6 trajectories stratified by admission IL-6 tercile. Predicted IL-6 values at each time point were obtained from a linear mixed-effects model and plotted across three time points: admission, 6-9 months, and 18-21 months. Participants were grouped according to their admission IL-6 tercile and mean predicted IL-6 trajectories were visualised using line plots with point estimates.
